# Association between serum 25-hydroxyvitamin D and vitamin D dietary supplementation and risk of all-cause and cardiovascular mortality among adults with hypertension

**DOI:** 10.1186/s12937-024-00914-8

**Published:** 2024-03-09

**Authors:** Haowen Ye, Yexin Li, Shaomin Liu, Xiaofang Zhang, Huanzhu Liang, Ying Wang, Ruxin Wang, Han Liu, Yun Wen, Chunxia Jing, Lihong Wang

**Affiliations:** 1 Department of Endocrinology and Metabolism, First Affiliated Hospital of Jinan University, Guangzhou, China; 2 Department of Public Health and Preventive Medicine, School of Medicine, Jinan University, No.601 Huangpu Ave West, Guangzhou, Guangdong 510632 China; 3 Guangdong Key Laboratory of Environmental Exposure and Health, Jinan University, Guangzhou, Guangdong 510632 China; 4 Department Clinical Experimental Center, First Affiliated Hospital of Jinan University, Guangzhou, China; 5 Department of Oncology Medilcal Center, The First People’s Hospital of Zhaoqing, Zhaoqing, China

**Keywords:** Serum 25 (OH)D, Vitamin D supplementation, Hypertension, NHANES, Mortality

## Abstract

**Background:**

The relationship between vitamin D status and mortality among adults with hypertension remains unclear.

**Methods:**

This prospective cohort study involved a sample of 19,500 adults with hypertension who participated in the National Health and Nutrition Examination Survey (NHANES) from 2001 to 2018. We utilized a weighted COX proportional hazard model to assess the association between vitamin D status and mortality. This statistical model calculates hazard ratios (HR) and their corresponding 95% confidence intervals (95% CI).

**Results:**

The study indicated that lower serum 25(OH)D concentration was associated with an increased risk of all-cause mortality among individuals with hypertension. Specially. Those with concentrations between 25.0 and 49.9 nmol/L (HR = 1.71, 95%CI = 1.22–2.40) and less than 25.0 nmol/L (HR = 1.97, 95%CI = 1.15–3.39) had higher hazard ratios for all-cause mortality. Individuals with hypertension who took vitamin D supplements had a lower risk of all-cause mortality, but not the risk of CVD mortality (HR 0.75, 95%CI 0.54–1.03), compared to those who did not supplement (HR = 0.76, 95%CI = 0.61–0.94). Subgroup analysis further revealed that vitamin D supplementation was associated with a reduced risk of all-cause mortality among individuals without diabetes (HR = 0.65, 95%CI = 0.52–0.81) and individuals without CVD (HR = 0.75, 95%CI = 0.58–0.97), and a decreased risk of CVD mortality among individuals without diabetes (HR = 0.63, 95%CI = 0.45–0.88) and without CVD (HR = 0.61, 95%CI = 0.40–0.92). Furthermore, higher-dose vitamin D supplementation was also associated with a greater reduction in all-cause mortality among hypertensive individuals, and there was the potential synergistic effect of combining normal-dose calcium and vitamin D supplementation, showing a superior effect on mortality compared to low-dose supplementation in adults with hypertension.

**Conclusions:**

This prospective cohort study demonstrated a significant association between lower serum 25 (OH)D concentration and increased all-cause mortality among adults with hypertension. Furthermore, the study found that vitamin D supplementation had a strong and significantly positive correlation with reduced all-cause and CVD mortality among hypertensive individuals without diabetes or CVD. This positive correlation suggests that vitamin D supplementation could potentially be an effective strategy to reduce the risk of mortality in this specific group of people.

**Supplementary Information:**

The online version contains supplementary material available at 10.1186/s12937-024-00914-8.

## Introduction

According to recent statistics, it was found that by 2019, approximately 52% of women and 43% of men worldwide had been diagnosed with hypertension [1, 2]. Despite improvements in diet and modern lifestyles, the prevalence of hypertension continues to rise, even affecting younger individuals [1–3]. This persistent and poorly controlled hypertension poses significant risks for cardiovascular disease and mortality, with impacts such as increased cardiac workload, vascular damage, blood clot formation, aneurysm development, and cerebrovascular events [4].

Serum 25-hydroxyvitamin D [25 (OH)D], which includes both 25 (OH)D2 and 25 (OH)D3, is the main form of vitamin D in the bloodstream and is used as an indicator to assess vitamin D levels [5]. Vitamin D deficiency is widespread, and the use of vitamin D supplements has become increasingly common [6–8]. Observational studies have shown that higher concentrations of serum 25 (OH)D are associated with a reduced blood pressure, lower risk of cardiovascular disease (CVD) and all-cause mortality in adults [9–14]. The presence of specific vitamin D receptors in cardiovascular tissue in basic experimental studies suggests a direct role of vitamin D in maintaining cardiovascular function [15]. Given the association between vitamin D and hypertension and CVD-related deaths, it is important to determine whether vitamin D is associated with mortality in hypertension, and whether the use of vitamin D supplements can improve mortality in hypertensive individuals. Nanri A et al. conducted a study on the correlation between dietary vitamin D intake and mortality in the Japanese and suggested that higher dietary vitamin D could reduce the risk of mortality in hypertension [16].In addition, the Honolulu Heart Program study found a significant association between low dietary vitamin D intake and higher overall mortality during 45 years of follow-up among middle-aged Japanese American men with hypertension, but not in those without hypertension [17]. Both studies indicate that higher dietary vitamin D intake is associated with lower mortality in individual with hypertension. Vitamin D supplements can provide more vitamin D than dietary intake, so whether additional vitamin D supplements can reduce mortality in individuals with hypertension. However, no studies have investigated the association between vitamin D supplementation and mortality in individual with hypertension.

Therefore, our objective is to investigate and analyze the association between serum 25 (OH)D levels, vitamin D supplementation, and mortality in adults with hypertension. This research aims to provide valuable insights and references for preventing mortality in adults with hypertension and determining the potential benefits of vitamin D supplements in this population.

## Research design and methods

### Study population

The National Health and Nutrition Examination Survey (NHANES), a study conducted by the Centers for Disease Control and Prevention (CDC) to evaluate the nutritional status of the U.S. population for preventive measures, encompasses data on population diet, health interviews, physical examinations and laboratory analyses. The NHANES protocol received approval from the Institutional Review Board of the National Center for Health Statistics, and all participants provided informed consent.

**Fig. 1:**
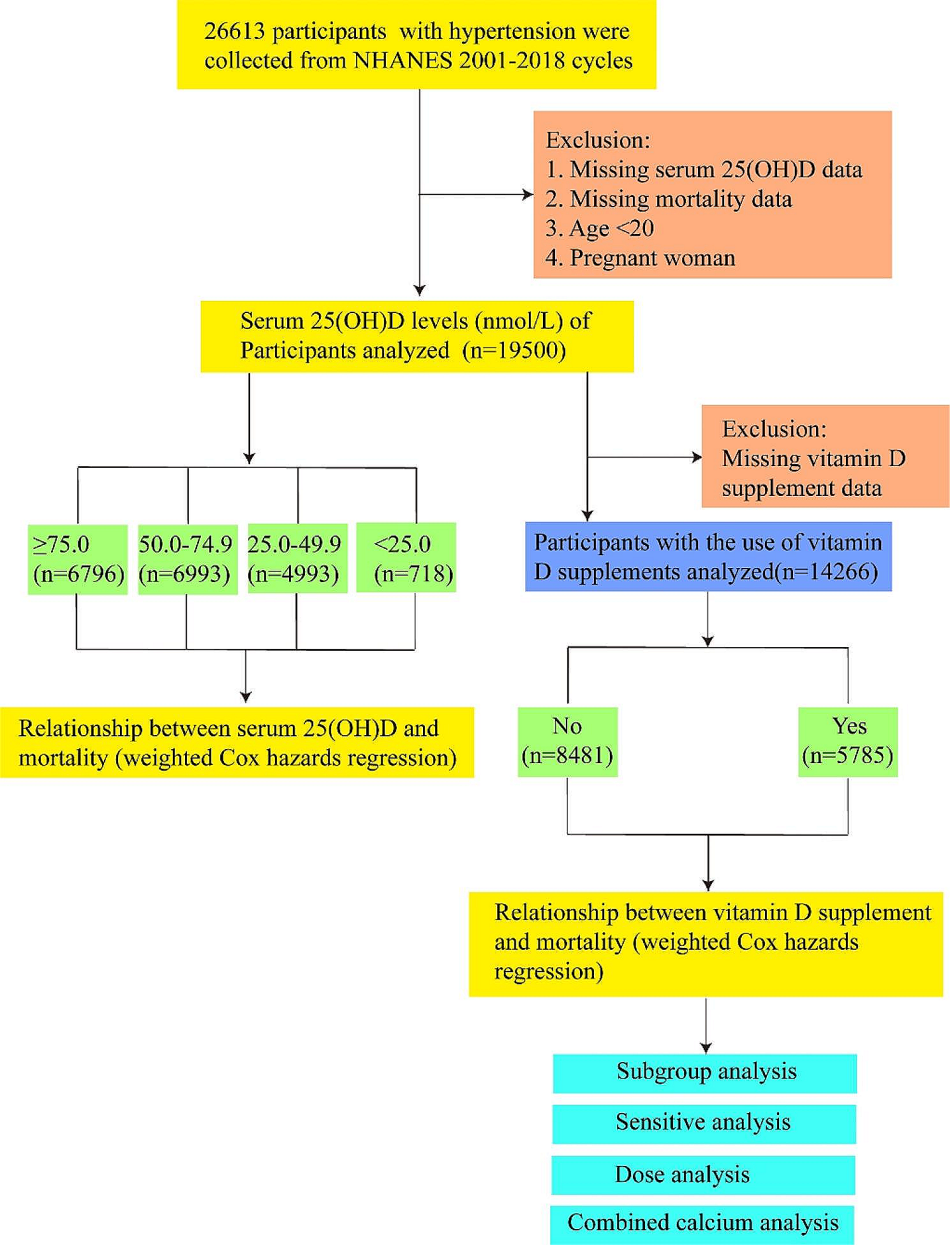
Flowchart of the study population

In the study, we acquired data from nine NHANES cycles spanning 2001 to 2018. Hypertension was defined as a self-reported physician diagnosis of hypertension, an average of three systolic blood pressure (SBP) ≥ 140 mmHg or an average of three diastolic blood pressure (DBP) ≥ 90 mmHg, and the use of antihypertensive drugs for primary hypertension. For a clinical point of view, considering the wide range of antihypertensive drugs used, such as beta blockers can be used for arrhythmia or heart failure, calcium channel blockers can be used for myocardial infarction or arrhythmia, we only used the prescription drug information from 2013 to 2018 because the prescription drug questionnaire before 2013 did not record the reason for medication use. A total of 26,613 participants with hypertension underwent screening. After excluding pregnant women, individuals under 20 years old, those with missing serum 25 (OH)D records, and incomplete death data, a total of 19,500 people were included in the study. The experimental design flow chart of this study is shown in Fig. 1.

### Measurement of serum 25 (OH)D concentrations

Blood samples were obtained from participants through venipuncture, and serum specimens were processed, stored, and shipped to the National Center for Environmental Health. Serum 25 (OH)D concentrations were measured using DiaSorin RIA kit (Stillwater MN) from 2001 to 2006 and a standardized liquid chromatography-tandem mass spectrometry (LC-MS/MS) method from 2007 to 2018. According to the suggestion of CDC in NHANES website, we employed a homologous regression equation to convert serum 25 (OH)D concentrations from 2001 to 2006 to facilitate subsequent analysis. The conversion formula of serum vitamin D concentration in NHANES 2001–2006 is as follows:

2001–2002: LC-MS/MS equivalent = 6.43435 + 0.95212*RIA original.

2003–2004: LC-MS/MS equivalent = 1.72786 + 0.98284*RIA original.

2005–2006: LC-MS/MS equivalent = 8.36753 + 0.97012*RIA original.

Total 25(OH)D, 25(OH)D3 and C3 epimer: 1 nmol/L = 0.40066 ng/mL (1 ng/mL = 2.4959 nmol/L).

### Vitamin D supplement use

Participants who reported no use of dietary supplements in the past 30 days were categorized as non-vitamin D supplement users, while those with total vitamin D supplement intake greater than 0 were considered vitamin D supplement users. or the period 2001–2006, total vitamin D supplementation in the past 30 days was calculated based on frequency, duration, and serving form, using the formula:

2001–2006: average daily supplement= (daily dosage* corresponding dosage form* days of use) /30.

### Mortality outcomes ascertainment

Mortality data was obtained from NHANES-linked National Death Index public access files through December 31, 2019. We used the International Statistical Classification of Disease, 10th Revision (ICD-10) codes to define the CVD deaths (I00-I09, I11, I13 and I20-I51). The participants’ death was considered as the endpoint for follow-up. Primary outcomes included all-cause mortality and CVD mortality, with specific attention to diseases of the heart, cerebrovascular disease, and hypertension flag, collectively considered as CVD death.

### Covariates

Demographic and lifestyle factors, including age, gender, race, education, ratio of family income to poverty (PIR), examination month, smoking status, alcohol consumption, duration of hypertension, antihypertensive therapy, physical activity, diet quality, calcium supplements, and comorbidities, were collected through detailed questionnaires during home interviews. Additionally, body mass index (BMI) data were obtained from physical examinations conducted at a mobile examination center. Considering the influence of light exposure on serum 25(OH)D concentration, we categorized it based on examination months: November 1 through April 30 and May 1 through October 31.

The Global Physical Activity Questionnaire, encompassing leisure, occupational, and travel activities, was utilized to gauge an individual’s physical activity. The Metabolic Equivalent (MET) value was calculated using the formula: MET (min/wk) = WET × weekly frequency × duration of each physical activity. Inactivity was defined as a MET value of less than 600 min/wk [18].

Diet quality was assessed using the 2015 Healthy Eating Index (HEI-2015), a tool that evaluates adherence to dietary guidelines, providing a comprehensive overview of dietary patterns and intake [19]. HEI-2015 scores below 50 and above 50 were categorized as inadequate and adequate, respectively [20].

Smoking status was classified as never smoker, ever smoker and current smoker according to whether they had smoked at least 100 cigarettes in life and whether they smoked cigarettes now. Those who had at least 12 alcohol drinks a year were considered as drinker. Low-to-moderate drinker was defined as alcohol drinks ≤ 2 drinks/day in men or ≤ 1 drinks/day in women. Heavy drinker was defined as alcohol drinks > 2 drinks/day in men or > 1 drinks/day in women. Cardiovascular disease (CVD) included congestive heart failure, coronary heart disease, angina, heart attack and stroke. Co-morbidities included diabetes, hypercholesterolemia, CVD, renal failure and cancer.

### Statistical analysis

Given the complex sampling design of NHANES, a meticulous approach to weighting the data was employed, following NHANES analysis guidelines. Sample weights, strata, and primary sampling units were utilized to account for the complex survey design. The data were combined and weighted using the wtmec2 year under the NHANES protocol.

According to the Endocrine Society Clinical Practice Guidelines, we divided serum 25 (OH)D concentrations into four groups: sufficient group (≥ 75.0 nmol/L), insufficient group (50.0-74.9 nmol/L), moderate deficiency group (25.0-49.9 nmol/L) and severe deficiency group (< 25.0 nmol/L) [21]. Considering several groups of vitamin D deficiency as adverse status, we used the sufficient group as the control group. COX proportional hazard method was employed to analyze the hazard ratio (HR) and 95% confidence intervals (95% CI) of all-cause mortality and CVD mortality in hypertension associated with serum 25 (OH)D concentration and vitamin D dietary supplementation.

Two multivariate model analyses were conducted: model 2 adjusted for age, gender, race, and survey period, while model 3 adjusted for all covariates. Additionally, a restricted cubic spline (RCS) model was employed to assess the nonlinear relationship between serum 25(OH)D concentration, vitamin D supplementation, and mortality. Univariate and multivariate weighted linear regression analyses were performed to investigate the impact of vitamin D supplementation on serum 25(OH)D levels.

Subgroup analysis was conducted according to gender (male and female), age (< 65 years and ≥ 65 years), BMI (< 25.0, 25.0-29.9 and ≥ 30.0), PIR (< 1.30, 1.30–3.49 and ≥ 3.50), duration of hypertension (≤ 10 years and > 10 years), diabetes (no and yes), hypercholesterolemia (no and yes), renal failure (no and yes), CVD (no and yes) and osteoporosis (no and yes). In addition, we also did sensitivity analyses. (1) Considering that mortality was related to physical activity and calcium supplements, we further adjusted them. (2) Participants who died within 1 year of follow-up was excluded. (3) Participants with a history of CVD was excluded. (4) Adults with osteoporosis were more likely to use vitamin D supplements, so we adjusted this variable further in the analysis of vitamin D supplementation and mortality. (5) Diet quality was adjusted.

Finally, we classified vitamin D supplementation by dose and calcium to further clarify the association between supplementation and mortality in hypertension. The doses were classified as 2000 IU/day (50 µg/day) and 4000 IU/day (100 µg/day) respectively. Based on supplement recommendations from most studies, we considered vitamin D < 400 IU/day (10 µg/day) and calcium < 600 mg/day to be low doses, and vitamin D ≥ 400 IU/day (10 µg/day) and calcium ≥ 600 mg/day to be normal doses [22].

All statistical analyses were conducted using R 4.2.2, and *p*-values < 0.05 were considered statistically significant.

## Results

### Baseline characteristics of participants

In the investigation of the correlation between serum 25 (OH)D and all-cause mortality, as well as CVD mortality in hypertension, a total of 19,500 participants were included, with 52.59% being female and a mean age of 58.8 years. The mean serum 25 (OH)D concentration, both before and after weighting, was 66.73 nmol/L and 71.32 nmol/L, respectively. Individuals with sufficient vitamin D were more likely to use vitamin D supplements (*n* = 3443/5785). Additional baseline data are presented in Table 1, and the weighted baseline information is available in S-Table 1.

**Table 1:** Baseline characteristics according to serum 25 (OH)D group

	Serum 25 (OH)D concentrations (nmol/L)						
	All	≥75.0	50.0-74.9	25.0-49.9	<25.0	*P*-value	
**N (%)**	19,500 (100.00)	6796 (34.85)	6993 (35.86)	4993 (25.61)	718 (3.68)		
**Gender (%)**						<0.001	
Male	9244 (47.41)	3035 (44.66)	3603 (51.52)	2326 (46.59)	280 (39.00)		
Female	10,256 (52.59)	3761 (55.34)	3390 (48.48)	2667 (53.41)	438 (61.00)		
**Age (years), mean (SD)**	58.8 (16.26)	62.4 (15.65)	58.2 (16.14)	55.3 (16.30)	54.2 (15.77)	<0.001	
**Race (%)**						0.000	
Mexican American	2650 (13.59)	552 (8.12)	1119 (16.00)	897 (17.97)	82 (11.42)		
Non-Hispanic White	9125 (46.79)	4387 (64.55)	3337 (47.72)	1286 (25.76)	115 (16.02)		
Non-Hispanic Black	4688 (24.04)	888 (13.07)	1364 (19.51)	1998 (40.02)	438 (61.00)		
Other Race	3037 (15.57)	969 (14.26)	1173 (16.77)	812 (16.26)	83 (11.56)		
**Education (%)**						<0.001	
Less than high school	5606 (28.79)	1528 (22.52)	2148 (30.76)	1711 (34.32)	219 (30.54)		
High school or equivalent	4723 (24.26)	1719 (25.34)	1649 (23.61)	1178 (23.63)	177 (24.69)		
College or above	9142 (46.95)	3537 (52.14)	3187 (45.63)	2097 (42.06)	321 (44.77)		
**PIR (%)**						<0.001	
<1.30	5651 (31.56)	1593 (25.45)	2020 (31.40)	1761 (38.70)	277 (41.91)		
1.30–3.49	6991 (39.05)	2473 (39.51)	2520 (39.17)	1735 (38.13)	263 (39.79)		
≥3.50	5261 (29.39)	2193 (35.04)	1893 (29.43)	1054 (23.16)	121 (18.31)		
**BMI (%)**						<0.001	
<25.0	4332 (22.75)	1898 (28.51)	1429 (20.93)	894 (18.37)	111 (16.16)		
25.0-29.9	6242 (32.79)	2363 (35.50)	2332 (34.15)	1379 (28.34)	168 (24.45)		
≥30.0	8465 (44.46)	2396 (35.99)	3068 (44.93)	2593 (53.29)	408 (59.39)		
**Examination month (%)**						<0.001	
November 1 through April 30	9173 (47.04)	2686 (39.52)	3224 (46.10)	2816 (56.40)	447 (62.26)		
May 1 through October 31	10,327 (52.96)	4110 (60.48)	3769 (53.90)	2177 (43.60)	271 (37.74)		
**Smoking (%)**						<0.001	
Never smoker	9869 (50.65)	3431 (50.52)	3553 (50.85)	2545 (51.02)	340 (47.35)		
Ever smoker	3682 (18.90)	981 (14.45)	1279 (18.31)	1200 (24.06)	222 (30.92)		
Current smoker	5933 (30.45)	2379 (35.03)	2155 (30.84)	1243 (24.92)	156 (21.73)		
**Drinking (%)**						<0.001	
Nondrinker	6279 (40.44)	2194 (39.83)	2194 (39.85)	1665 (42.24)	226 (39.58)		
Low-to-moderate drinker	4787 (30.83)	1937 (35.17)	1714 (31.14)	1016 (25.77)	120 (21.02)		
Heavy drinker	4460 (28.73)	1377 (25.00)	1597 (29.01)	1261 (31.99)	225 (39.40)		
**Duration of hypertension (%)**						<0.001	
≤3 years	2724 (24.29)	843 (20.64)	962 (26.62)	804 (29.30)	115 (23.47)		
4–10 years	2761 (25.25)	935 (22.89)	968 (26.78)	729 (26.57)	129 (26.33)		
>10 years	5448 (49.83)	2307 (56.47)	1684 (46.60)	1211 (44.13)	246 (50.20)		
**Antihypertensive therapy (%), yes**	11,760 (87.74)	4463 (90.71)	4075 (87.28)	2791 (84.50)	431 (84.34)	<0.001	
**The use of vitamin D** **dietary supplements (%)**						0.000	
No	8481 (59.45)	1578 (31.43)	3095 (64.18)	3278 (85.36)	530 (90.91)		
Yes	5785 (40.55)	3443 (68.57)	1727 (35.82)	562 (14.64)	53 (9.09)		
**Co-morbidities (%)**							
Diabetes	4164 (22.09)	1368 (20.87)	1432 (21.17)	1161 (24.04)	203 (28.88)	<0.001	
Hypercholesterolemia	9041 (52.03)	3462 (55.37)	3254 (52.55)	2045 (47.55)	280 (44.30)	<0.001	
CVD	3941 (20.35)	1433 (21.23)	1336 (19.25)	1002 (20.20)	170 (23.74)	0.004	
Renal failure	1091 (5.61)	463 (6.83)	305 (4.37)	278 (5.59)	45 (6.29)	< 0.001	
Cancer	2665 (13.68)	1275 (18.78)	850 (12.17)	477 (9.56)	63 (8.79)	< 0.001	

**Abbreviation**: PIR, ratio of family income to poverty; BMI, body mass index (calculated as weight in kilograms divided by height in meters squatted); CVD, cardiovascular disease

CVD included congestive heart failure, coronary heart disease, angina, heart attack and stroke

### Serum 25 (OH)D concentration and mortality

Over a mean follow-up of 8.8 years, 4481 adults with hypertension experienced mortality, including 1940 CVD deaths. As detailed in Table 2, serum 25 (OH)D concentration in participants with hypertension exhibited associations with all-cause and CVD mortality in models 1 and 2. Compared to the reference group (sufficient group, ≥ 75.0 nmol/L), all-cause mortality increased in those with serum 25 (OH)D deficiency (25.0-49.9 nmol/L, HR = 1.71, 95%CI = 1.22–2.40; <25.0 nmol/L, HR = 1.97, 95%CI = 1.15–3.39) after adjusting for multiple variables. However, lower serum 25 (OH)D concentration in hypertension was not significantly associated with CVD mortality (*P* for trend = 0.12).

**Table 2:** HRs (95%CIs) for mortality according to serum 25 (OH)D concentrations among hypertensive participants

	Serum 25 (OH)D concentrations (nmol/L)				
	≥75.0	50.0-74.9	25.0-49.9	<25.0	P for trend
**All-cause mortality**					
Deaths, yes (%) (all = 4481)	1462/6796 (21.51)	1645/6993 (23.52)	1192/4993 (23.87)	182/718 (25.35)	
Model 1 HR (95% CI) *P*-value	1.00	1.00 (0.91–1.09) 0.91	1.20 (1.05–1.36) 0.01	1.73 (1.40–2.13) < 0.001	< 0.001
Model 2 HR (95% CI) *P*-value	1.00	1.19 (1.09–1.30) < 0.001	1.79 (1.59–2.02) < 0.001	3.04 (2.44–3.79) < 0.001	< 0.001
Model 3 HR (95% CI) *P*-value	1.00	1.17 (0.95–1.45) 0.14	1.71 (1.22–2.40) 0.002	1.97 (1.15–3.39) 0.01	< 0.001
**CVD mortality**					
Deaths, yes (%) (all = 1940)	655/6796 (9.64)	686/6993 (9.81)	521/4993 (10.43)	78/718 (10.86)	
Model 1 HR (95% CI) *P*-value	1.00	0.96 (0.84–1.10) 0.56	1.24 (1.04–1.49) 0.02	1.65 (1.21–2.25) 0.002	0.01
Model 2 HR (95% CI) *P*-value	1.00	1.15 (1.01–1.32) 0.04	1.87 (1.55–2.27) < 0.001	2.91 (2.04–4.13) < 0.001	< 0.001
Model 3 HR (95% CI) *P*-value	1.00	1.06 (0.75–1.49) 0.75	1.71 (0.94–3.14) 0.08	1.42 (0.70–2.91) 0.33	0.12

**Abbreviations**: CVD, cardiovascular disease

**Model 1**: Unadjusted; **Model 2**: Adjusted for age, sex, race and survey cycle; **Model 3**: Adjusted for age, gender, race, survey cycle, education, PIR, BMI, smoking, drinking, during of hypertension, antihypertensive therapy, vitamin D supplementation and co-morbidities

### Assessment of the correlation between vitamin D supplementation and serum 25 (OH)D concentration

To investigate the effects of vitamin D dietary supplementation, univariate and multivariate weighted linear regression analyses were conducted to assess the correlation between vitamin D supplementation and serum 25 (OH)D concentration (S-Table 2). Univariate linear regression indicated a low positive association between vitamin D supplementation and serum 25 (OH)D concentration in participants with hypertension (*β* = 0.09, 95%CI = 0.03–0.15). After adjusting for multiple covariables, this positive correlation remained stable (*β* = 0.14, 95%CI = 0.09–0.18).

### Vitamin D dietary supplementation and mortality

Given the association between lower serum 25 (OH)D concentration and increased mortality, the impact of vitamin D supplementation on mortality in participants with hypertension was explored using weighted COX regression. Baseline characteristics according to the use of vitamin D supplements are detailed in S-Table 3 and S-Table 4 before and after weighting, respectively. Vitamin D supplement non-users had an average serum 25 (OH)D concentration of 55.36 nmol/L, while users had 83.45 nmol/L after weighting.

As presented in Table 3, after adjusting for multiple variables, participants with hypertension who used vitamin D supplements exhibited reduced all-cause mortality compared to non-users (HR = 0.76, 95%CI = 0.61–0.94). However, there was no significant difference in CVD mortality (*P*-value = 0.07).

**Table 3:** HRs (95%CIs) for mortality according to the use of vitamin D supplement among participants with hypertension

	Vitamin D supplementation			
	No	Yes		*P*-value
**All-cause mortality**				
Deaths, yes (%)	1872/8481 (22.07)	1067/5785 (18.44)		
Model 1 HR (95% CI)	1.00	1.20 (1.08–1.32)		< 0.001
Model 2 HR (95% CI)	1.00	0.65 (0.59–0.72)		< 0.001
Model 3 HR (95% CI)	1.00	0.76 (0.61–0.94)		0.01
**CVD mortality**				
Deaths, yes (%)	799/8481 (9.42)	452/5785 (7.81)		
Model 1 HR (95% CI)	1.00	1.19 (1.05–1.34)		0.32
Model 2 HR (95% CI)	1.00	0.63 (0.55–0.71)		< 0.001
Model 3 HR (95% CI)	1.00	0.75 (0.54–1.03)		0.07

**Abbreviations**: CVD, cardiovascular disease

**Model 1**: Unadjusted; **Model 2**: Adjusted for age, sex, race and survey cycle; **Model 3**: Adjusted for age, gender, race, survey cycle, education, PIR, BMI, smoking, drinking, during of hypertension, antihypertensive therapy and co-morbidities

### Detection of nonlinear relationship

**Fig. 2:**
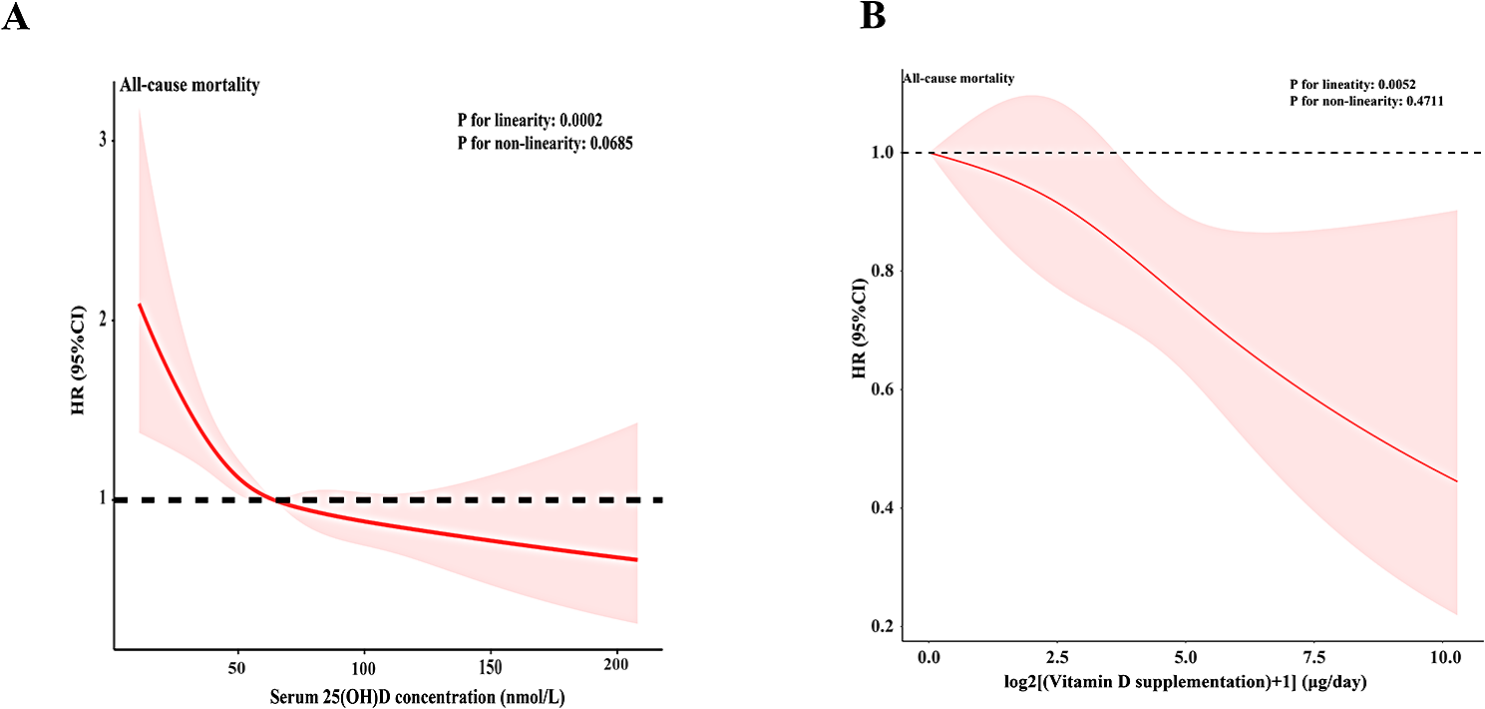
Association between 25 (OH)D concentration (**A**) as well as vitamin D supplements (**B**) and all-cause in adults with hypertension. Adjusted for age, gender, race, survey cycle, education, PIR, BMI, smoking, drinking, during of hypertension, medication, vitamin D supplements and co-morbidities. The red solid lines and pink areas represent the estimates and their corresponding 95% confidence intervals, respectively

A restricted cubic spline (RCS) model was employed to examine the relationship between serum 25 (OD)D concentration, its supplementation, and mortality in participants with hypertension. Serum 25 (OH)D concentration demonstrated a linear relationship with all-cause mortality (P for linearity = 0.0002, P for nonlinearity = 0.0685, Fig. 2A). Similarly, there was a linear relationship between the use of vitamin D supplements and mortality, including all-cause mortality (P for linearity = 0.0052, P for nonlinearity = 0.4711, Fig. 2B).

### Subgroup and sensitivity analyses

**Fig. 3:**
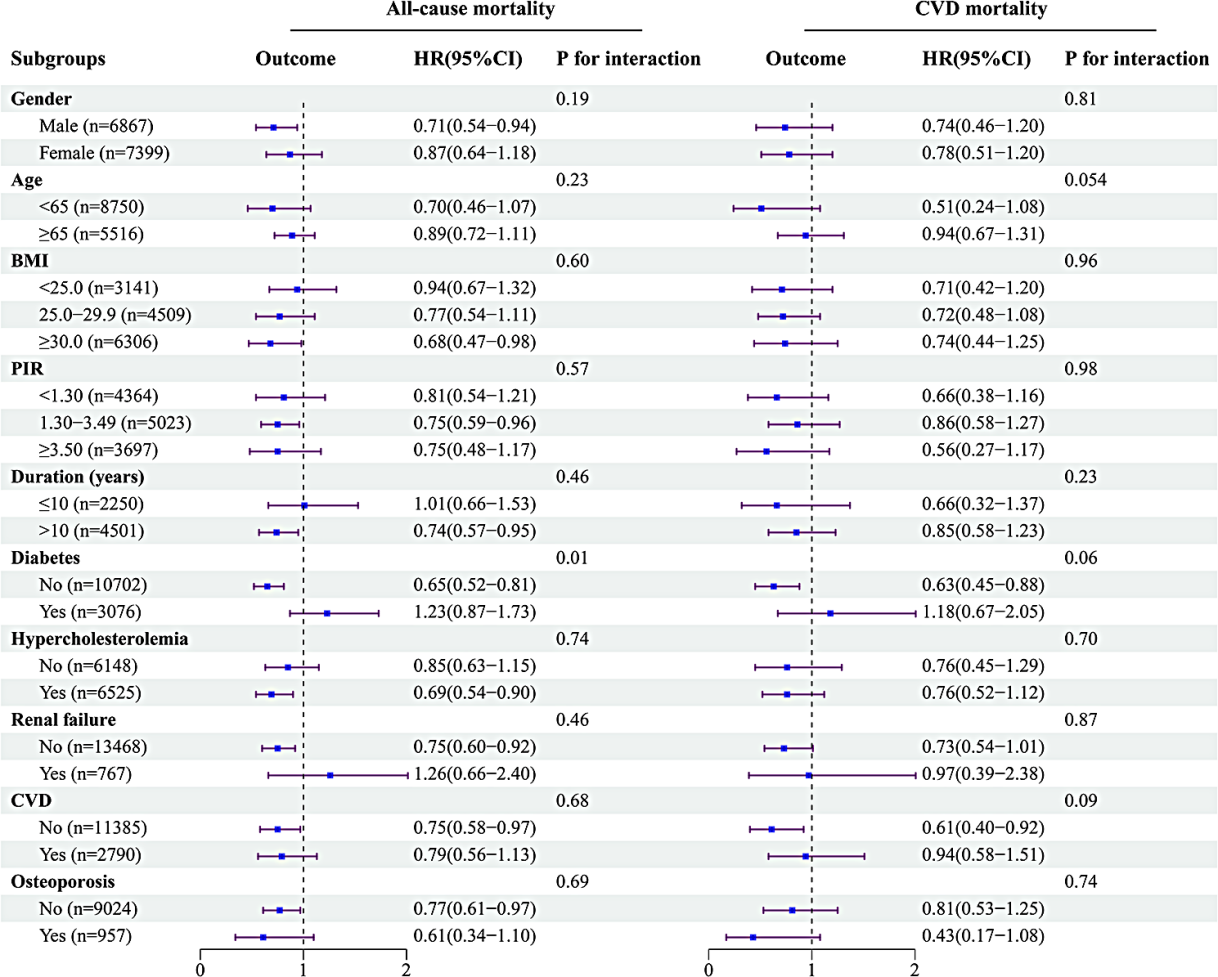
Forest plots of subgroup analyses of vitamin D supplements and all-cause and CVD mortality in adults with hypertension. Age, gender, race, survey cycle, education, PIR, BMI, smoking, drinking, during of hypertension, medication, vitamin D supplements and co-morbidities were adjusted except the variable itself.

Subgroup analyses revealed consistent increases in all-cause mortality in participants with hypertension using vitamin D supplements across most subgroups (Fig. 3). Notably, the use of vitamin D supplements in those without a history of diabetes or CVD was associated not only with reduced all-cause mortality (without diabetes, HR = 0.65, 95%CI = 0.52–0.81; without CVD, HR = 0.75, 95%CI = 0.58–0.97), but also with reduced CVD mortality (without diabetes, HR = 0.63, 95%CI = 0.45–0.88; without CVD, HR = 0.61, 95%CI = 0.40–0.92). Additionally, there was a significant interaction of all-cause mortality between vitamin D supplementation and a history of diabetes (P for interaction < 0.05).

In three sensitivity analyses, the robust association between serum 25 (OH)D concentration (especially in the group 25.0-49.9 nmol/L) and all-cause mortality persisted in participants with hypertension (S-Table 5). Further adjustments for a history of osteoporosis, physical activity, diet quality, and exclusion of participants who died within 1 year of follow-up confirmed the statistically significant correlation between vitamin D supplementation and all-cause mortality in participants with hypertension (S-Table 6).

In grouping the population into different doses of vitamin D supplementation, high doses were more strongly associated with low all-cause mortality (≥ 50 µg/day, HR = 0.50, 95%CI = 0.32–0.79; ≥100 µg/day, HR = 0.29, 95%CI = 0.12–0.69) in adults with hypertension, and also significantly with CVD mortality (≥ 50 µg/day, HR = 0.39, 95%CI = 0.18–0.88; ≥100 µg/day, HR = 0.20, 95%CI = 0.06–0.65) (S-Table 7).

### Effect of vitamin D combined with calcium supplementation on mortality in adults with hypertension

Considering that vitamin D is commonly used in combination with calcium supplements, the population was divided into four groups for analysis: non-supplementation, normal dose vitamin D + low dose calcium, normal dose calcium + low dose vitamin D, and normal dose vitamin D + normal dose calcium. Multiple COX regression models indicated that the group receiving normal dose vitamin D combined with normal dose calcium supplementation was significantly associated with reduced all-cause mortality (HR = 0.68, 95%CI = 0.51–0.92) and CVD mortality (HR = 0.51, 95%CI = 0.34–0.77) in adults with hypertension (S-Table 8).

## Discussion

In this prospective cohort study of adults with hypertension in the United States, our findings reveal a significant inverse relationship between serum 25(OH)D concentration and all-cause mortality. Additionally, the use of vitamin D supplements is associated with a reduced risk of all-cause mortality in adults with hypertension, and notably, this association extends to both all-cause and CVD mortality for individuals without diabetes or pre-existing cardiovascular conditions. Moreover, our investigation into the impact of vitamin D supplement dosage and the concomitant use of calcium supplements on mortality in the individual with hypertension demonstrates a strong association between high-dose vitamin D supplements, combined calcium supplements, and lower all-cause and CVD mortality. To the best of our knowledge, this is the first study to explore the link between vitamin D supplementation and mortality in adults with hypertension.

Currently, prevailing research primarily focuses on the general population, and there is no consensus regarding the association between serum 25(OH)D concentration, vitamin D supplements, and mortality. Most studies posit a connection between low serum 25(OH)D concentration and elevated mortality, while the impact of vitamin D supplementation on mortality remains inconclusive [10, 14, 23–27]. However, within the context of personalized precision therapy, our study suggests that the relationship between vitamin D and mortality may exhibit greater stability and significance within specific populations. Two studies supported our findings, including one conducted outside the United States. Park D et al. demonstrated that a low level of serum 25(OH)D increases the risk of all-cause and specific death in Koreans with hypertension [11]. Similarly, Zhao G et al. analyzed NHANES data from 2001 to 2004 and concluded that serum 25(OH)D concentration is associated with all-cause and CVD mortality in adults with hypertension [28]. While our study aligns with these findings, the observed weaker association with CVD mortality may stem from differences in the study population and the duration of follow-up.

Given the previous evidence linking low serum 25(OH)D levels with higher mortality in individuals with hypertension, our study sought to determine whether vitamin D supplementation could mitigate this risk. Contrary to findings from numerous prospectively studies suggesting that vitamin D supplementation does not significantly impact overall mortality, our study, focusing specifically on the hypertensive population, reveals consistent results with two studies on dietary vitamin D intake and mortality in hypertension [17, 29–31]. Moreover, our findings suggest that the benefits of vitamin D supplementation may be more pronounced in individuals with hypertension lacking a history of diabetes or CVD. This extends beyond the impact of antihypertensive medications, affecting both all-cause and CVD mortality. Patients with prediabetes who received high doses of vitamin D had a reduced risk of developing T2DM [32], while T2DM use had no significant effect on glycemic control or cardiovascular risk [33, 34]. Vitamin D supplementation did not improve the risk of mortality in critically ill patients [35, 36]. Michael W et al. concluded that vitamin D supplementation did not reduce the risk of stroke or heart attack in patients with known CVD [37]. These results suggest the ineffectiveness of vitamin D supplementation in hypertension with diabetes or CVD. Compared to population-wide studies, no consistent results were observed for the benefit of vitamin D supplementation on population-wide mortality, possibly due to the presence of a history of diabetes or CVD. This may be because diabetes and CVD have a complex and severe impact on mortality, and vitamin D supplementation does not ameliorate this effect. The effect of vitamin D supplementation on mortality in hypertension seems to be unrelated to blood pressure control, but may be related to immune regulation, anti-inflammatory and improvement of blood lipids [38–43]. In addition, subgroup analysis showed that vitamin D supplement use was more strongly associated with lower all-cause mortality in hypertension with high BMI and hyperlipidemia. It has previously been reported to reduce waist-to-hip ratio and increase adiponectin and leptin levels in obese people [44, 45]. Mechanistically, vitamin D supplementation can improve the parameters of high-fat induced inflammation and obesity [46, 47].In conclusion, our study suggests that male, BMI ≥ 30, duration > 10 years, hypercholesterolemia, non-osteoporosis, non-diabetes, non-renal failure and non-CVD may be indications of vitamin D supplementation in adults with hypertension.

While previous studies have recommended daily vitamin D doses ranging from 800 to 1000 IU (20–25 µg) daily as optimal, our study suggests that high-dose vitamin D supplementation, specifically 4000 IU (100 µg) daily, is associated with reduced all-cause and CVD mortality in adults with hypertension [22]. This suggests that high dose vitamin D supplementation not only with reduced all-cause mortality in hypertension, but also with reduced CVD mortality, which provides evidence for the benefits of high dose vitamin D supplementation in adults with hypertension. Importantly, our study found no evidence to suggest harm from high doses of vitamin D supplementation in this population. Since vitamin D supplementation are mostly used in combination with calcium, we divided hypertensive population into four groups for analysis, in order to further explore the role of vitamin D supplementation in the mortality of hypertension population. A study has reported that whether alone or combination, vitamin D supplementation and calcium have no significant effect on all-cause and CVD mortality in the whole population [48]. However, this conclusion appears to be reversed in the meta-analysis [49]. This may be related to population-specific disease, as a review comparing the association between vitamin D supplementation and mortality in people with different disease states suggests that vitamin D supplementation has different effects on disease-specific and patient-oriented outcomes [37]. Our study demonstrated that normal dose vitamin D supplementation in combination with normal dose calcium was associated with better mortality reduction than with low dose vitamin D supplementation or low dose calcium among adults with hypertension. Vitamin D effectively acts as a calcium supplement by promoting calcium absorption. When using vitamin D supplementation, the appropriate addition of calcium can have a better benefit. It is noteworthy, however, that the combined use of vitamin D and calcium may increase the risk of kidney stones and potentially promote vascular calcification, necessitating further exploration of the efficacy of single versus combined use [50, 51]. More studies are needed in the future to reveal the efficacy of single use versus combined use.

The physiological roles of vitamin D, primarily mediated through binding to intracellular vitamin D receptors and influencing various cellular functions, include the regulation of the cell cycle, the renin-angiotensin-aldosterone system (RAAS), vascular endothelial growth factor production, and anti-fibrotic effects [52–55]. Although low serum 25(OH)D levels are linked to an increased risk of coronary heart disease, heart failure, and atrial fibrillation, our study found no evidence supporting the efficacy of vitamin D supplementation in preventing coronary heart disease, heart failure, or atrial fibrillation [56–60]. There is no evidence that vitamin D supplementation is associated with atrial fibrillation. Moreover, it not only plays an important anti-inflammatory role by reducing inflammatory cytokine storms, but also reduces oxidative stress and improves vascular endothelial function [61]. In combination with this study, the protective effect of vitamin D on blood vessels and its anti-inflammatory and antioxidant effects may be the important reasons for the reduction of CVD mortality in hypertensive individual without the history of diabetes or CVD.

Our research carries several strengths. As far as we are aware, this is the first study with a large sample size and a long follow-up period that examines the impact of serum 25 (OH)D concentration and the use of vitamin D supplements on mortality in individuals with hypertension. We have taken into account multiple confounding factors, increasing the reliability and accuracy of our findings. Our study not only investigates the effects of serum 25 (OH)D concentration on mortality in hypertensive individuals but also explores the potential benefits of vitamin D in combination with calcium supplementation.

Our study also has some limitations. Firstly, because the NHANES database is not longitudinal, it is not possible to infer a causal relationship between vitamin D concentration and its supplementation on all-cause mortality in hypertension. Secondly, vitamin D concentration was measured only once at baseline, which has some limitations for assessing its levels. Finally, residual or unknown confounder effects due to measurement errors and unmeasured variables (such as psychosocial stress and genetic predisposition) cannot be excluded.

## Conclusion

Overall, this study found that lower serum 25 (OH)D concentration was associated with increased all-cause mortality in adults with hypertension, and that adults with hypertension who took vitamin D supplements had a lower risk of all-cause mortality. Vitamin D supplementation in hypertensive individual without diabetes or CVD was strongly associated with lower risk of all-cause and CVD mortality. Our study suggests that hypertensive individuals without diabetes or CVD are more likely to benefit from vitamin D supplementation, and may provide evidence for the individualized use of vitamin D supplements in clinic.

### Electronic supplementary material

Below is the link to the electronic supplementary material.

Supplementary Material 1

## Data Availability

The original data were retrieved from NHANES database (https://www.cdc.gov/nchs/nhanes/index.htm).

## Abbreviations

25(OH)D: Serum 25-hydroxyvitamin D
CVD: Cardiovascular disease
NHANES: The National Health and Nutrition Examination Survey
CDC: Centers for Disease Control and Prevention
SBP: Systolic blood pressure
DBP: Diastolic blood pressure
LC-MS/MS: Liquid chromatography-tandem mass spectrometry
PIR: Ratio of family income to poverty
HR: Hazard ratio
95% CI: 95% confidence intervals
RCS: Restricted cubic spline
