# Supplementary material for: Association between serum 25-hydroxyvitamin D and vitamin D dietary supplementation and risk of all-cause and cardiovascular mortality among adults with hypertension
Source: Nutr J. 2024 Mar 9;23:33. doi: 10.1186/s12937-024-00914-8 (PMC10924411; doi:10.1186/s12937-024-00914-8)
Supplement: Supplementary file 1 — Supplementary Material 1 [file 12937_2024_914_MOESM1_ESM.docx]

Catalogue

[**S-Table 1 Baseline characteristics according to serum 25 (OH) D group after weighting** 2](#_Toc151798250)

[**S-Table 2 Association between vitamin D supplementation and serum 25 (OH) D concentration in hypertension** 4](#_Toc151798251)

[**S-Table 3 Baseline characteristics according to the use of vitamin D dietary supplements before weighting** 5](#_Toc151798252)

[**S-Table 4 Baseline characteristics according to the use of vitamin D dietary supplements after weighting** 7](#_Toc151798253)

[**S-Table 5 Sensitive analysis between serum 25 (OH) D concentration and mortality in adults with hypertension** 9](#_Toc151798254)

[**S-Table 6 Sensitive analysis between vitamin D supplementation and mortality in adults with hypertension** 10](#_Toc151798255)

[**S-Table 7 The relationship between vitamin D supplementation and mortality according to the dose of vitamin D supplementation** 11](#_Toc151798256)

[**S-Table 8 The relationship between vitamin D supplementation in combination with calcium and mortality according to the dose of vitamin D and calcium supplementation** 12](#_Toc151798257)

**S-Table 1 Baseline characteristics according to serum 25 (OH) D group after weighting**

|  | **Serum 25(OH) D concentrations (nmol/L)** | | | | | |
| --- | --- | --- | --- | --- | --- | --- |
|  | **All** | **≥75.0** | **50.0-74.9** | **25.0-49.9** | **<25.0** | **P-value** |
| **N (%)** | 81127667.4 (100.0) | 33769342.4 (41.6) | 29100269.2 (35.9) | 16196723.4 (20.0) | 2061332.3 (2.5) |  |
| **Gender (%)** |  |  |  |  |  | <0.001 |
| Female | 43529236.0 (53.7) | 19162181.0 (56.7) | 14088811.0 (48.4) | 8966420.8 (55.4) | 1311823.2 (63.6) |  |
| **Age (years), mean (SD)** | 55.60 (16.19) | 58.52 (15.95) | 54.30 (16.05) | 52.34 (15.95) | 51.62 (15.81) | <0.001 |
| **Race (%)** |  |  |  |  |  | <0.001 |
| Mexican American | 4674715.2 (5.8) | 949350.9 (2.8) | 1953582.4 (6.7) | 1597112.4 (9.9) | 174669.5 (8.5) |  |
| Non-Hispanic White | 57662044.5 (71.1) | 28474687.9 (84.3) | 20781953.5 (71.4) | 7743702.6 (47.8) | 661700.6 (32.1) |  |
| Non-Hispanic Black | 10183966.4 (12.6) | 1720504.4 (5.1) | 2923517.8 (10.0) | 4520791.4 (27.9) | 1019152.8 (49.4) |  |
| Other Race | 8606941.4 (10.6) | 2624799.2 (7.8) | 3441215.6 (11.8) | 2335117.1 (14.4) | 205809.4 (10.0) |  |
| **Education (%)** |  |  |  |  |  | <0.001 |
| Less than high school | 15225703.0 (18.8) | 4882363.8 (14.5) | 5799517.9 (19.9) | 4001019.5 (24.7) | 542801.8 (26.4) |  |
| High school or equivalent | 20594879.6 (25.4) | 8741546.6 (25.9) | 7217364.1 (24.8) | 4137245.7 (25.6) | 498723.2 (24.2) |  |
| College or above | 45229332.1 (55.8) | 20112674.5 (59.6) | 16069181.4 (55.2) | 8029671.8 (49.7) | 1017804.5 (49.4) |  |
| **PIR (%)** |  |  |  |  |  | <0.001 |
| <1.30 | 16347125.9 (21.6) | 5197730.0 (16.5) | 5823759.1 (21.3) | 4627117.7 (30.8) | 698519.2 (36.6) |  |
| 1.30-3.49 | 28258946.7 (37.3) | 11582077.9 (36.8) | 10170110.4 (37.2) | 5685779.4 (37.9) | 820979.1 (43.0) |  |
| ≥3.50 | 31102766.5 (41.1) | 14675330.6 (46.7) | 11350673.7 (41.5) | 4687801.2 (31.3) | 388961.0 (20.4) |  |
| **BMI (%)** |  |  |  |  |  | <0.001 |
| <25.0 | 18384713.2 (23.1) | 9437010.4 (28.4) | 5749112.5 (20.2) | 2875931.6 (18.1) | 322658.6 (16.4) |  |
| 25.0-29.9 | 25459032.9 (32.0) | 11634471.2 (35.1) | 9226381.4 (32.3) | 4106209.0 (25.9) | 491971.2 (25.0) |  |
| ≥30.0 | 35707361.4 (44.9) | 12114016.5 (36.5) | 13547769.8 (47.5) | 8890425.1 (56.0) | 1155150.1 (58.6) |  |
| **Examination month (%)** |  |  |  |  |  |  |
| May 1 through October 31 | 47078573.6 (58.0) | 21851946.9 (64.7) | 17030326.1 (58.5) | 7425149.6 (45.8) | 771151.0 (37.4) | <0.001 |
| **Smoking (%)** |  |  |  |  |  | <0.001 |
| Never smoker | 40821600.9 (50.4) | 17008949.7 (50.4) | 14559124.2 (50.0) | 8253339.0 (51.0) | 1000188.0 (48.5) |  |
| Ever smoker | 15693435.4 (19.4) | 5326437.1 (15.8) | 5601364.7 (19.3) | 4127360.9 (25.5) | 638272.7 (31.0) |  |
| Current smoker | 24558710.2 (30.3) | 11402305.3 (33.8) | 8931501.0 (30.7) | 3802032.2 (23.5) | 422871.6 (20.5) |  |
| **Drinking (%)** |  |  |  |  |  | <0.001 |
| Nondrinker | 23014029.4 (34.2) | 9293127.8 (32.6) | 7950851.2 (33.2) | 5153903.9 (39.2) | 616146.5 (36.9) |  |
| Low-to-moderate drinker | 22648083.9 (33.7) | 10619445.6 (37.3) | 8109236.6 (33.9) | 3562280.9 (27.1) | 357120.7 (21.4) |  |
| Heavy drinker | 21581769.3 (32.1) | 8581155.7 (30.1) | 7866184.4 (32.9) | 4439022.3 (33.7) | 695407.0 (41.7) |  |
| **Duration of hypertension (%)** |  |  |  |  |  | <0.001 |
| ≤3 years | 11494244.2 (25.8) | 4234405.9 (21.7) | 4103717.7 (27.9) | 2855943.9 (32.2) | 300176.6 (21.8) |  |
| 4–10 years | 12147196.5 (27.3) | 4901837.1 (25.1) | 4414065.5 (30.0) | 2491797.3 (28.1) | 339496.6 (24.7) |  |
| >10 years | 20826415.9 (46.8) | 10384405.0 (53.2) | 6195256.5 (42.1) | 3511104.5 (39.6) | 735649.8 (53.5) |  |
| **Antihypertensive therapy (%), yes** | 45999698.7 (86.3) | 20437359.0 (88.5) | 15717993.0 (85.7) | 8661610.3 (82.9) | 1182736.3 (84.2) | <0.001 |
| **The use of Vitamin D supplements (%)** | 25916904.0 (44.2) | 16602979.7 (66.7) | 7126652.6 (36.0) | 1988746.9 (16.2) | 198524.8 (12.0) | <0.001 |
| **Co-morbidities (%)** |  |  |  |  |  |  |
| Diabetes | 13830004.4 (17.6) | 5356466.8 (16.4) | 4678414.6 (16.6) | 3276817.8 (20.9) | 518305.2 (25.6) | <0.001 |
| Hypercholesterolemia | 37377846.3 (51.2) | 16727700.9 (54.0) | 13166139.9 (50.5) | 6698259.0 (47.4) | 785746.5 (43.1) | <0.001 |
| CVD | 13763724.9 (17.1) | 5791455.0 (17.2) | 4586795.6 (15.9) | 2916936.9 (18.1) | 468537.4 (22.8) | 0.03 |
| Renal failure | 3508056.0 (4.3) | 1705660.0 (5.1) | 922667.5 (3.2) | 749986.8 (4.6) | 129741.6 (6.3) | <0.001 |
| Cancer | 11589005.0 (14.3) | 6200609.1 (18.4) | 3562225.3 (12.3) | 1637277.3 (10.1) | 188893.3 (9.2) | <0.001 |

**Abbreviation:** serum 25 (OH)D, Serum 25-hydroxyvitamin D; PIR, ratio of family income to poverty; BMI, body mass index (calculated as weight in kilograms divided by height in meters squatted); CVD, cardiovascular disease.

CVD included congestive heart failure, coronary heart disease, angina, heart attack and stroke.

**S-Table 2 Association between vitamin D supplementation and serum 25 (OH) D concentration in hypertension by linear regression**

|  | **Serum 25 (OH) D concentration (nmol/L)** | | |
| --- | --- | --- | --- |
|  | **Model 1**  **(*β*, 95%CI)** | **Model 2**  **(*β*, 95%CI)** | **Model 3**  **(*β*, 95%CI)** |
| **Vitamin D supplementation (μg/day)** | 0.09 (0.03, 0.15) | 0.07 (0.02, 0.12) | 0.14 (0.09, 0.18) |

**Model 1:** Unadjusted; **Model 2**: Adjusted for age, gender, race and survey cycle; **Model 3**: Adjusted for age, gender, race, survey cycle, examination month, education, PIR, BMI, smoking, drinking, during of hypertension, antihypertensive therapy and co-morbidities.

**S-Table 3 Baseline characteristics according to the use of vitamin D dietary supplements before weighting**

|  | **Vitamin D supplementation** | | |  |
| --- | --- | --- | --- | --- |
|  | **All** | **No** | **Yes** | **P-value** |
| **N (%)** | 14266 (100.00) | 8481 (59.45) | 5785 (40.55) |  |
| **Serum 25 (OH)D**  **concentration (nmol/L), mean (SD)** | 66.75 (29.01) | 55.36 (22.34) | 83.45 (29.57) | 0.000 |
| **Gender (%)** |  |  |  | <0.001 |
| Male | 6867 (48.14) | 4504 (53.11) | 2363 (40.85) |  |
| Female | 7399 (51.86) | 3977 (46.89) | 3422 (59.15) |  |
| **Age (years), mean (SD)** | 57.81 (16.30) | 54.69 (16.67) | 62.37 (14.59) | <0.001 |
| **Race (%)** |  |  |  | <0.001 |
| Mexican American | 1994 (13.98) | 1416 (16.70) | 578 (9.99) |  |
| Non-Hispanic White | 6279 (44.01) | 3282 (38.70) | 2997 (51.81) |  |
| Non-Hispanic Black | 3603 (25.26) | 2431 (28.66) | 1172 (20.26) |  |
| Other Race | 2390 (16.75) | 1352 (15.94) | 1038 (17.94) |  |
| **Education (%)** |  |  |  | <0.001 |
| Less than high school | 4260 (29.91) | 3122 (36.87) | 1138 (19.70) |  |
| High school or equivalent | 3392 (23.81) | 2083 (24.60) | 1309 (22.65) |  |
| College or above | 6593 (46.28) | 3262 (38.53) | 3331 (57.65) |  |
| **PIR (%)** |  |  |  | <0.001 |
| <1.30 | 4364 (33.35) | 3071 (39.45) | 1293 (24.40) |  |
| 1.30-3.49 | 5023 (38.39) | 2946 (37.84) | 2077 (39.20) |  |
| ≥3.50 | 3697 (28.26) | 1768 (22.71) | 1929 (36.40) |  |
| **BMI (%)** |  |  |  | <0.001 |
| <25.0 | 3141 (22.51) | 1776 (21.49) | 1365 (23.98) |  |
| 25.0-29.9 | 4509 (32.31) | 2601 (31.47) | 1908 (33.52) |  |
| ≥30.0 | 6306 (45.18) | 3887 (47.04) | 2419 (42.50) |  |
| **Smoking (%)** |  |  |  | <0.001 |
| Never smoker | 7231 (50.73) | 4055 (47.85) | 3176 (54.95) |  |
| Ever smoker | 2887 (20.25) | 2220 (26.20) | 667 (11.54) |  |
| Current smoker | 4136 (29.02) | 2199 (25.95) | 1937 (33.51) |  |
| **Drinking (%)** |  |  |  | <0.001 |
| Nondrinker | 4481 (39.48) | 2518 (37.92) | 1963 (41.68) |  |
| Low-to-moderate drinker | 3416 (30.09) | 1789 (26.94) | 1627 (34.54) |  |
| Heavy drinker | 3454 (30.43) | 2334 (35.15) | 1120 (23.78) |  |
| **Duration of hypertension (%)** |  |  |  | <0.001 |
| ≤3 years | 2250 (24.80) | 1261 (28.05) | 989 (21.61) |  |
| 4-10 years | 2321 (25.58) | 1255 (27.92) | 1066 (23.29) |  |
| >10 years | 4501 (49.61) | 1979 (44.03) | 2522 (55.10) |  |
| **Antihypertensive therapy (%), yes** | 8536 (87.03) | 4529 (84.37) | 4007 (90.25) | <0.001 |
| **Co-morbidities (%)** |  |  |  |  |
| Diabetes | 3076 (22.33) | 1797 (21.83) | 1279 (23.06) | 0.09 |
| Hypercholesterolemia | 6525 (51.49) | 3359 (47.12) | 3166 (57.11) | <0.001 |
| CVD | 2790 (19.68) | 1598 (18.97) | 1192 (20.73) | 0.01 |
| Renal failure | 767 (5.39) | 410 (4.84) | 357 (6.19) | 0.001 |
| Cancer | 1860 (13.05) | 831 (9.81) | 1029 (17.81) | <0.001 |

**Abbreviation**: serum 25 (OH)D, Serum 25-hydroxyvitamin D; PIR, ratio of family income to poverty; BMI, body mass index (calculated as weight in kilograms divided by height in meters squatted); CVD, cardiovascular disease.

CVD included congestive heart failure, coronary heart disease, angina, heart attack and stroke.

**S-Table 4 Baseline characteristics according to the use of vitamin D dietary supplements after weighting**

|  | **Vitamin D supplementation** | | |  |
| --- | --- | --- | --- | --- |
|  | **All** | **No** | **Yes** | **P-value** |
| **N (%)** | 59870799 (100.0) | 32756079 (56.8) | 25916904 (44.2) |  |
| **Serum 25 (OH)D**  **concentration (nmol/L), mean (SD)** | 71.79 (29.20) | 60.30 (23.43) | 86.32 (29.30) | <0.001 |
| **Gender (%)** |  |  |  | <0.001 |
| Female | 30998640.5 (52.8) | 15398580.8 (47.0) | 15600059.7 (60.2) |  |
| **Age (years), mean (SD)** | 55.00 (16.26) | 50.78 (16.17) | 60.34 (14.73) | <0.001 |
| **Race (%)** |  |  |  | <0.001 |
| Mexican American | 3717998.1 (6.3) | 2679365.8 (8.2) | 1038632.3 (4.0) |  |
| Non-Hispanic White | 40580954.3 (69.2) | 20807075.8 (63.5) | 19773878.5 (76.3) |  |
| Non-Hispanic Black | 7840373.5 (13.4) | 5421396.8 (16.6) | 2418976.7 (9.3) |  |
| Other Race | 6533657.1 (11.1) | 3848240.6 (11.7) | 2685416.5 (10.4) |  |
| **Education (%)** |  |  |  | <0.001 |
| Less than high school | 11624749.9 (19.8) | 8314750.8 (25.4) | 3309999.1 (12.8) |  |
| High school or equivalent | 14576594.4 (24.9) | 8827450.7 (27.0) | 5749143.7 (22.2) |  |
| College or above | 32423715.5 (55.3) | 15577982.6 (47.6) | 16845733.0 (65.0) |  |
| **PIR (%)** |  |  |  | <0.001 |
| <1.30 | 12573522.6 (23.0) | 8818563.2 (28.9) | 3754959.3 (15.6) |  |
| 1.30-3.49 | 20234556.0 (37.0) | 11460414.8 (37.5) | 8774141.2 (36.4) |  |
| ≥3.50 | 21852511.8 (40.0) | 10277130.0 (33.6) | 11575381.8 (48.0) |  |
| **BMI (%)** |  |  |  | <0.001 |
| <25.0 | 13024150.0 (22.6) | 6926588.5 (21.6) | 6097561.5 (23.9) |  |
| 25.0-29.9 | 18310485.3 (31.8) | 9732041.5 (30.3) | 8578443.7 (33.6) |  |
| ≥30.0 | 26299417.9 (45.6) | 15416955.6 (48.1) | 10882462.3 (42.6) |  |
| **Smoking (%)** |  |  |  | <0.001 |
| Never smoker | 29623957.5 (50.5) | 15557298.8 (47.5) | 14066658.7 (54.3) |  |
| Ever smoker | 11829872.9 (20.2) | 9034380.5 (27.6) | 2795492.3 (10.8) |  |
| Current smoker | 17170704.8 (29.3) | 8142803.8 (24.9) | 9027901.0 (34.9) |  |
| **Drinking (%)** |  |  |  | <0.001 |
| Nondrinker | 16270451.7 (33.5) | 8557192.7 (32.1) | 7713259.0 (35.1) |  |
| Low-to-moderate drinker | 16091477.5 (33.1) | 7715594.4 (28.9) | 8375883.1 (38.2) |  |
| Heavy drinker | 16245720.0 (33.4) | 10381945.0 (38.9) | 5863774.9 (26.7) |  |
| **Duration of hypertension (%)** |  |  |  | <0.001 |
| ≤3 years | 9565870.9 (25.6) | 5044487.2 (29.7) | 4521383.7 (22.1) |  |
| 4-10 years | 10287551.7 (27.5) | 5055859.7 (29.7) | 5231692.0 (25.6) |  |
| >10 years | 17585762.3 (47.0) | 6900410.0 (40.6) | 10685352.3 (52.3) |  |
| **Antihypertensive therapy (%), yes** | 34062501.8 (86.0) | 16108526.0 (82.2) | 17953975.8 (89.7) | <0.001 |
| **Co-morbidities (%)** |  |  |  |  |
| Diabetes | 10044587.1 (17.7) | 5284759.0 (16.6) | 4759828.2 (19.1) | 0.001 |
| Hypercholesterolemia | 26848986.8 (50.8) | 12625707.0 (45.6) | 14223279.8 (56.7) | <0.001 |
| CVD | 9745128.4 (16.7) | 5083931.0 (15.6) | 4661197.4 (18.1) | 0.002 |
| Renal failure | 2410968.4 (4.1) | 1151898.3 (3.5) | 1259070.1 (4.9) | 0.001 |
| Cancer | 8214003.1 (14.0) | 3158649.7 (9.7) | 5055353.5 (19.5) | <0.001 |

**Abbreviation**: serum 25 (OH)D, Serum 25-hydroxyvitamin D; PIR, ratio of family income to poverty; BMI, body mass index (calculated as weight in kilograms divided by height in meters squatted); CVD, cardiovascular disease.

CVD included congestive heart failure, coronary heart disease, angina, heart attack and stroke.

**S-Table 5 Sensitive analysis between serum 25 (OH) D concentration and mortality in adults with hypertension**

|  | **Serum 25 (OH) D concentrations (nmol/L)** | | | | |
| --- | --- | --- | --- | --- | --- |
|  | **≥75.0** | **50-74.9** | **25.0-49.9** | **<25.0** | ***P* for trend** |
| **All-cause mortality** |  |  |  |  |  |
| Model a  HR (95% CI) *P*-value | 1.00 | 1.22 (0.97-1.54) 0.09 | 1.84 (1.29-2.63) <0.001 | 2.19 (1.26-3.82) 0.01 | <0.001 |
| Model b  HR (95% CI) *P*-value | 1.00 | 1.15 (0.92-1.44) 0.21 | 1.71 (1.20-2.43) 0.003 | 1.88 (1.06-3.32) 0.03 | 0.002 |
| Model c  HR (95% CI) *P*-value | 1.00 | 0.92 (0.67-1.25) 0.16 | 1.71 (1.09-2.68) 0.03 | 1.79 (0.85-3.78) 0.16 | 0.04 |
| **CVD mortality** |  |  |  |  |  |
| Model a  HR (95% CI) *P*-value | 1.00 | 1.07 (0.75-1.53) 0.70 | 1.77 (0.93-3.34) 0.08 | 1.61 (0.80-3.24) 0.18 | 0.10 |
| Model b  HR (95% CI) *P*-value | 1.00 | 1.05 (0.74-1.49) 0.79 | 1.82 (0.97-3.40) 0.06 | 1.48 (0.71-3.11) 0.30 | 0.10 |
| Model c  HR (95% CI) *P*-value | 1.00 | 0.91 (0.51-1.60) 0.73 | 1.88 (0.79-4.48) 0.15 | 1.27 (0.47-3.45) 0.64 | 0.28 |

**Model a:** Further adjusted the physical activity and calcium supplements; **Model b**: Excluding the participants who died within 1 year of follow-up; **Model c**: Excluding the participants with a history of CVD.

**S-Table 6 Sensitive analysis between vitamin D supplementation and mortality in adults with hypertension**

|  | **The use of Vitamin D supplements** | |
| --- | --- | --- |
|  | **All-cause mortality** | **CVD mortality** |
| Model a  HR (95% CI) *P*-value | 0.78 (0.62, 0.98) 0.03 | 0.77 (0.55, 1.07) 0.12 |
| Model b  HR (95% CI) *P*-value | 0.72 (0.52, 0.998) 0.049 | 0.63 (0.41, 0.98) 0.04 |
| Model c  HR (95% CI) *P*-value | 0.72 (0.53, 0.98) 0.04 | 0.60 (0.37, 0.97) 0.04 |

**Model a**: Excluding the participants who died within 1 year of follow-up; **Model b**: Further adjusted physical activity, diet quality; **Model c:** Further adjusted osteoporosis.

**S-Table 7 The relationship between vitamin D supplementation and mortality according to the dose of vitamin D supplementation**

|  | **The use of Vitamin D supplements [HR (95% CI) P-value]** | |
| --- | --- | --- |
|  | **All-cause mortality** | **CVD mortality** |
| **Dose 1** |  |  |
| <50 μg (2000 IU)/day (n=4773) | 0.80 (0.65, 0.98) 0.03 | 0.81 (0.59, 1.11) 0.19 |
| ≥50 μg (2000 IU)/day (n=1012) | 0.50 (0.32, 0.79) 0.003 | 0.39 (0.18, 0.88) 0.02 |
| **Dose 2** |  |  |
| <100 μg (4000 IU)/day (n=5471) | 0.78 (0.63, 0.96) 0.02 | 0.77 (0.56, 1.06) 0.10 |
| ≥100 μg (4000 IU)/day (n=314) | 0.29 (0.12, 0.69) 0.005 | 0.20 (0.06, 0.65) 0.008 |

**S-Table 8 The relationship between vitamin D supplementation in combination with calcium and mortality according to the dose of vitamin D and calcium supplementation**

|  | **Supplements [HR (95% CI) P-value]** | | | | |
| --- | --- | --- | --- | --- | --- |
|  | **No** | **Normal dose vitamin D + low dose calcium** | **Normal dose calcium + low dose vitamin D** | **Normal dose vitamin D + normal dose calcium** | ***P* for trend** |
| **N** | 8481 | 2498 | 133 | 1224 |  |
| **All-cause mortality** | 1.00 | 0.80 (0.63-1.02) 0.07 | 0.97 (0.56-1.68) 0.91 | 0.68 (0.51-0.92) 0.01 | 0.01 |
| **CVD mortality** | 1.00 | 0.87 (0.59-1.28) 0.49 | 1.05 (0.52-2.12) 0.89 | 0.51 (0.34-0.77) 0.001 | 0.002 |

vitamin D <400 IU/day (10 μg/day) and calcium <600 mg/day were considered to be low doses, and vitamin D ≥400 IU/day (10 μg/day) and calcium ≥600 mg/day were considered to be normal doses.
